# Characterisation of tropomyosin and paramyosin as vaccine candidate molecules for the poultry red mite, *Dermanyssus gallinae*

**DOI:** 10.1186/s13071-016-1831-8

**Published:** 2016-10-12

**Authors:** Harry W. Wright, Kathryn Bartley, John F. Huntley, Alasdair J. Nisbet

**Affiliations:** Moredun Research Institute, Pentlands Science Park, Bush Loan, Edinburgh, Midlothian EH26 0PZ Scotland, UK

**Keywords:** *Dermanyssus gallinae*, Paramyosin, Tropomyosin, Poultry red mite, Vaccine, Allergen

## Abstract

**Background:**

*Dermanyssus gallinae* is the most economically important haematophagous ectoparasite in commercial egg laying flocks worldwide. It infests the hens during the night where it causes irritation leading to restlessness, pecking and in extreme cases anaemia and increased cannibalism. Due to an increase in the occurrence of acaricide-resistant *D. gallinae* populations, new control strategies are required and vaccination may offer a sustainable alternative to acaricides. In this study, recombinant forms of *D. gallinae* tropomyosin (Der g 10) and paramyosin (Der g 11) were produced, characterised and tested as vaccine candidate molecules.

**Methods:**

The *D. gallinae* paramyosin (Der g 11) coding sequence was characterised and recombinant versions of Der g 11 and *D. gallinae* tropomyosin (Der g 10) were produced. Hens were immunised with the recombinant proteins and the resulting antibodies were fed to *D. gallinae* and mite mortality evaluated. Sections of mites were probed with anti- Der g 11 and Der g 10 antibodies to identify the tissue distribution of these protein in *D. gallinae.*

**Results:**

The entire coding sequence of *Der g 11* was 2,622 bp encoding 874 amino acid residues. Immunohistochemical staining of mite sections revealed that Der g 10 and Der g 11 were located throughout *D. gallinae* tissues. In phylogenetic analyses of these proteins both clustered with orthologues from tick species rather than with orthologues from astigmatid mites. Antibodies raised in hens against recombinant forms of these proteins significantly increased *D. gallinae* mortality, by 19 % for Der g 10 (*P* < 0.001) and by 23 % for Der g 11 (*P* = 0.009) when fed to the mites using an in vitro feeding device.

**Conclusions:**

This study has shown that Der g 10 and Der g 11 were located ubiquitously throughout *D. gallinae* and that antibodies raised against recombinant versions of these proteins can be used to significantly increase *D. gallinae* mortality in an in vitro feeding assay. When comparing archived data for all recombinant and native proteins assessed as vaccines using this in vitro feeding assay, Der g 10 and Der g 11 ranked highly and performed better than some of the pools of native proteins.

## Background

The poultry red mite (PRM), *Dermanyssus gallinae* (De Geer), is a blood-feeding ectoparasite that infests many bird species. From both an economic and welfare perspective, PRM is the most important ectoparasite affecting egg-laying hens worldwide [1, 2]. In 2005 it was estimated that €130 million was lost annually due to PRM in Europe [3] and it is likely that losses now far exceed that level. These mites reside in crevices of the furniture and machinery in the poultry house and emerge in darkness to feed, for short periods of time, on the blood of hens [4, 5]. Heavy infestations of *D. gallinae* of more than 200,000 mites per hen can result in anaemia, irritation, restlessness, feather pecking and, in extreme cases, death and increased levels of cannibalism [5, 6]. In addition, *D. gallinae* is becoming an important occupational health issue for poultry workers exposed to PRM bites and allergens [7]. Spraying of poultry facilities with chemical acaricides is the most widely-used method of control [8]. However, there is an increasing incidence of resistance to acaricides in *D. gallinae* [9–11] and the potential for accumulation of acaricide residues in eggs and poultry meat [12, 13] has resulted in a need for safer alternatives for the control of PRM. Several alternative methods of control are available or are in development, for example plant or fungal extracts, essential oils and vaccination [8].

Vaccination has shown promise for the control of a number of haematophagous parasites of livestock: The commercially-available Barbervax® vaccine contains native *Haemonchus contortus* gut antigens and has been shown to significantly reduce clinical disease in sheep during field use in Australia (reviewed in [14]). In addition, the ectoparasite vaccines TickGARD™ and Gavac™, were developed with the recombinant protein Bm86 as the protective antigen against the tropical cattle tick *Rhipicephalus microplus* in Australia and South America, respectively [15]. Bm86 homologues have since been fully characterised in several other tick species (*Rhipicephalus annulus*, *R. decoloratus*, *Haemaphysalis longicornis* and *Hyalomma marginatum marginatum* [16, 17]) but not in other members of the Acari. To date no conserved orthologues of the Bm86 antigen have been identified in transcriptomic data of *D. gallinae* [18, 19]. Therefore, other vaccine candidates are required for the development of a vaccine strategy for PRM control. One approach used for *D. gallinae* vaccine candidate identification has been the rational selection of antigens based upon their inferred orthology with promising vaccine candidates in other species [20]. The mite allergens tropomyosin (group 10 allergen) and paramyosin (group 11 allergen) [21] are microfilament proteins that facilitate the interaction between actin with myosin and troponin during filament assembly and contraction. Tropomyosin is ubiquitously found in eukaryotic cells and several tissue-specific isoforms exist [22, 23], whereas paramyosin is an invertebrate-specific component of the thick filament of muscles [24]. Tropomyosins are potent mite allergens [21] and both paramyosin and tropomyosin have shown promising results as vaccine candidates against a range of ecto- and endo-parasites including the trematode *Schistosoma japonica*, the filarioid nematode *Acanthocheilonema viteae* and the tick *Haemaphysalis longicornis* [25–28].

Tropomyosin has been characterised in a number of mite species including *Metaseiulus occidentalis* (94 and 97 % amino acid identity to *D. gallinae* tropomyosin for isoforms 1 (accession XP_003745222) and 2 (accession XM_003745175), respectively), the house dust mites (HDM) *Dermatophagoides pteronyssinus* (85 % identity), *D. farinae* (86 % identity) [29, 30] and sheep scab mites *Psoroptes ovis* (86 % identity) [21]. There are also several tick species with orthologues such as *R. microplus* (89 % identity) and *H. longicornis* (88 % identity) [31]. Recombinant tropomyosin from *H. longicornis* was used to immunise rabbits and, following tick feeding on vaccinated animals, significant reductions (*P* < 0.05) in tick engorgement weights (19.4 %), oviposition (49.5 %), egg mass (14.7 %) and egg hatching rates (100 %) were seen [27].

Paramyosin has also previously been identified as a potential vaccine candidate for parasite control: The native form of paramyosin from *S. japonicum* was used to immunise mice and, following challenge with the parasite, the adult worm burden in immunised mice compared to control mice was reduced by up to 86 % [25]. Immunisation of BALB/c mice with a recombinant cocktail vaccine containing *S. japonicum* paramyosin fragments reduced worm burden and liver-stage eggs by up to 40 and 78 %, respectively following challenge [28].

The aim of the work described here was to investigate the potential of recombinant versions of *D. gallinae* tropomyosin (Der g 10) and paramyosin (Der g 11), to control the ectoparasite *D. gallinae* when used to immunise hens.

## Methods

### *Dermanyssus gallinae* collection, extraction of total RNA and cDNA synthesis

Mixed stage and gender *D. gallinae* were collected from a commercial egg production unit and were snap frozen in liquid nitrogen within 4 h of collection. RNA was extracted and isolated using TRIZOL® reagent (Invitrogen, Carlsbad, United States) according to the manufacturer’s protocol. After DNaseI treatment and subsequent purification using RNeasy spin columns (Qiagen, Hilden, Germany), mRNA was purified from total RNA using the Poly(A)Purist™ kit (Ambion, Waltham, United States). Rapid amplification of cDNA ends (RACE)-ready cDNA was generated using the SMART™ RACE cDNA Amplification Kit (Clontech, Paris, France) using the manufacturer’s protocols.

### Characterisation of the genes encoding Der g 11 and Der g 10

A partial transcriptomic sequence representing the paramyosin gene *Der g 11* was identified in a *D. gallinae* Roche 454 sequencing dataset [20]. 5’ and 3’ RACE reactions were performed to amplify the remaining sections of coding sequence. RACE products were ligated into pGEM®-T Easy vector (Promega, Madison, United States) and DNA sequencing performed (Eurofins MWG Operon) to confirm the coding sequences (CDS). The full CDS of paramyosin was amplified using gene-specific oligonucleotide primers (forward, 5'-TTC GGA TCC GAT GGA GGC CAT CAA GAA TAA GAT G-3' and reverse, 5'-AGT GCG GCC GCG TAT CCG GTA AGC TCG GCG AA-3') and employing RACE-ready cDNA as the template with Advantage® 2 PCR polymerase (Clontech). The resulting paramyosin CDS was named *Der g 11* in accordance with systematic allergen nomenclature [32] and deposited in the NCBI database under accession number LT555400. The CDS of the *D. gallinae* tropomyosin gene (*Der g 10,* AM167555) was previously determined [31].

A homology search (Blastx) was performed using the NCBI nr database to identify homologues of *Der g 10* and *Der g 11*. The amino acid sequences of the top 20 hits for *Der g 11* and *Der g 10* were aligned using T-coffee (Version_11.00.8cbe486) [33, 34], which generated a neighbour-joining tree drawn according to relationships inferred by the multiple sequence alignment. The neighbour joining tree was bootstrapped 1,000 times using ClustalW and viewed in the package MEGA 6.06 [35] (6140226), the degree of bootstrap was shown only if above 50 %.

### Expression and purification of Der g 10 and Der g 11 as recombinant proteins

The full CDS of *Der g 11* and that of *Der g 10* were subcloned into pET-SUMO (Invitrogen) and pET-22b(+) (Novagen, Madison, United States) vectors, respectively in frame with the vector-encoded polyhistidine tags. Plasmid DNA was transformed into *Esherichia coli* BL21-CodonPlus® (DE3)-RIL competent cells (Stratagene, La Jolla, United States) and recombinant protein expression induced with 1 mM IPTG. Recombinant Der g 11 and Der g 10 were purified from inclusion bodies by nickel column affinity chromatography using HisTrap™ HP columns in the presence of 8 M urea (GE Healthcare) then dialysed against 20 mM phosphate buffer, 0.5 M NaCl, 2 M urea, pH 7.4. The Pierce BCA™ assay (Thermo Scientific, Waltham, United States) with bovine serum albumin standards were used to estimate the protein concentration. Eluted proteins were separated by electrophoresis on a 4–12 % NuPAGE® Novex Bis-Tris Gel (Invitrogen) and bands at the appropriate molecular weight for each protein were excised and analysed using MALDI-ToF spectroscopy to confirm their identity (Moredun Proteomics Unit).

### Generation of immunoglobulin specific for recombinant Der g 10 and Der g 11

Female Blackrock hens were injected, into the breast muscle, with either recombinant Der g 11 or Der g 10 (2 hens for each protein). Each dose comprised 75 μg of recombinant protein, 200 μg of QuilA adjuvant and PBS in a final volume of 300 μl. Booster injections were administered 2 and 4 weeks later. Sera and eggs were collected pre-immunisation, for use as a negative controls for Western blotting and in the feeding assay, and 2 weeks after the third immunisation (week 6), when the antibody level against the target proteins was predicted to be at a maximum. IgY was purified from the egg yolks using the Eggstract kit (Promega) following the manufacturer’s instructions and was stored at -20 °C. IgY concentration was determined using the Pierce BCA™ assay (Thermo Scientific). Equal amounts (3 mg/ml) of purified IgY from individual hens were pooled according to group and time.

All procedures were carried out in accordance with the UK Animals (Scientific Procedures) Act 1986. The ethics committee at Moredun Research Institute ratified the experimental design (08/09).

### Immunoblotting of *D. gallinae* extracts, recombinant Der g 10 and Der g 11

Proteins were extracted from freshly collected *D. gallinae* and fractionated into soluble, membrane-associated, integral-membrane and insoluble proteins as previously described [36]. Each protein fraction (~3 μg total protein per fraction) and recombinant protein (3 μg) was loaded onto a 4–12 % NuPAGE® Novex Bis-Tris gel (Invitrogen) and then separated by electrophoresis. The proteins were electroblotted onto nitrocellulose membranes and probed with a pool of IgY previously purified from egg yolk was diluted 1:200 with Tris-buffered saline (TBS) to ~30 μg/ml and the blot was developed as described in Bartley et al. [37].

### Immunolocalisation of Der g 10 and Der g 11 in sections of *D. gallinae*

Five micron sections of *D. gallinae* were immobilised onto glass slides, using the protocol described in Bartley et al. [37]. Sections were probed with primary antibodies generated in mice against *Blomia tropicalis* paramyosin (Blo t 11) [38] and *D. pteronyssinus* tropomyosin (Der p 10) [21]. These antibodies were previously shown to cross-react with Der g 10 and Der g 11 in Western blots (data not shown).

The immunolocalisation protocol was as described in Bartley et al. [39] with the following differences: the primary antibodies were diluted 1:200 with TBS for the mouse monoclonal IgG anti-Blo t 11, whereas the mouse polyclonal IgG anti-Der p 10 was diluted 1:1000. Bound antibodies were detected with a Rabbit anti-mouse-IgG/HRP conjugate (Dako, Glostrup, Denmark; P0260).

### Effects of consuming IgY specific for Der g 10 and Der g 11 on *D. gallinae* mortality

IgY antibodies raised in hens against either Der g 10 and Der g 11 were fed to pre-conditioned *D. gallinae* using the in vitro blood-feeding system described in Wright et al. [36] and the mortality of mites following feeding was monitored. Briefly, three groups of mites were fed with a blood meal prepared by mixing 2 ml fresh heparinised blood (36 USP units heparin per ml blood) obtained from *D. gallinae*-naive Lohmann Brown hens with 1 ml (6 mg) of a pooled IgY, previously described, purified from egg yolk collected pre-immunisation (control group) or 2 weeks after the third immunisation (week 6) with Der g 10 or Der g 11 (test groups). This experiment was repeated on three occasions using 10 in vitro chambers per experimental group. Approximately 200 *D. gallinae* were added to each chamber and 300 μl of blood containing IgY was added to the reservoir of each chamber. The chambers were incubated at 40 °C in darkness with a relative humidity of 75 % overnight.

Mites that had fed during the overnight incubation were separated into individual wells of a 96-well microtitre plate (Greiner bio-one, Kremsmunster, Austria), sealed with AeraSeal™ film (Sigma-Aldrich, St. Louis, United States; A9224-50EA,) and mortality was measured every 24 h for a 4-day period. The mite mortality data were fitted to a simple logistic regression model (SLRM), which individually compared the mortality from both test groups (Der g 10 and Der g 11-immunised) with that of the controls (immunised with adjuvant only) over the course of the experiment.

## Results

### Characterisation and phylogenetic relationships of Der g 10 and Der g 11

**Fig. 1:**
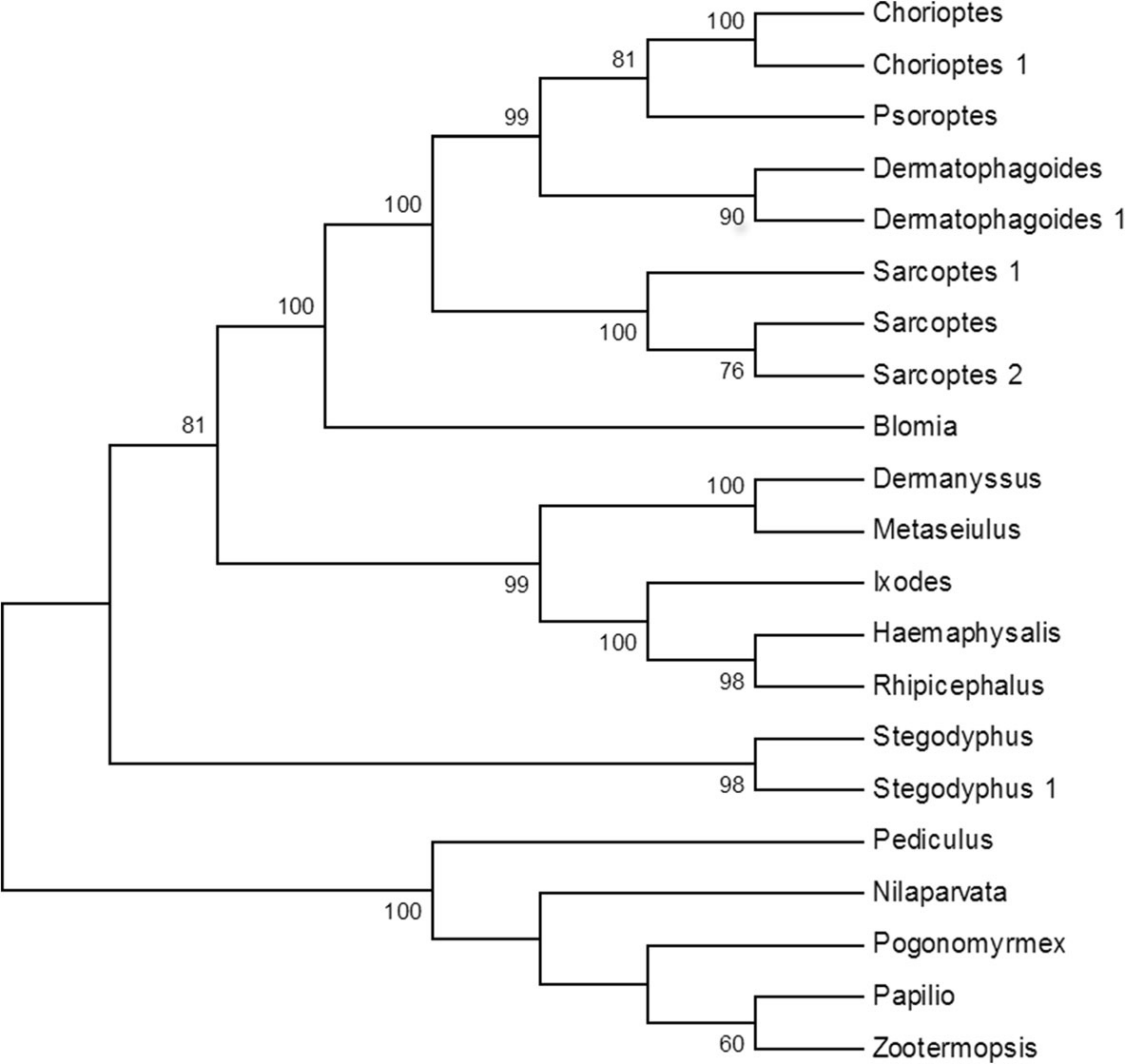
Relationships between selected sequences of Der g 11 from different invertebrate species. The neighbour-joining tree was bootstrapped 1,000 times using Clustal X and the resulting tree viewed with TreeView. Each node is annotated with a figure indicating the degree of bootstrap support for each branch. Abbreviations and accession numbers are as follows: *Dermanyssus* (*D. gallinae*), *Metaseiulus* (*M. occidentalis*; XP_003744495.1), *Rhipicephalus* (*R.* (*Boophilus*) *microplus*; AAO20875.1), *Haemaphysalis* (*H. longicornis*; AFR32950.1), *Ixodes* (*I. scapularis*; XP_002407289.1), *Sarcoptes* (*Sarcoptes scabiei*; ABV54632.1), *Sarcoptes* 1 (*S. scabiei*; ACC65584.1), *Dermatophagoides* (*D. farinae*; AIO08864.1), *Sarcoptes* 2 (*S. scabiei*; AAK01181.1), *Psoroptes* (*P. ovis*; CAJ38271), *Chorioptes* (*Chorioptes panda*; ACB30406.1), *Dermatophagoides* 1 (*D. farinae*; AAO73464.1), *Chorioptes* 1 (*C. texanus*; ABK54038.1), *Blomia*; *Blomia tropicalis* (Q8MUF6.1), *Stegodyphus* (*Stegodyphus mimosarum*; KFM81308.1), *Stegodyphus* 1 (*S. mimosarum*; KFM67302.1), *Pediculus* (*P. humanus corporis*; XP_002432355.1), *Zootermopsis* (*Zootermopsis nevadensis*; KDR08790.1), *Nilaparvata* (*Nilaparvata lugens*; AGI96992.1), *Pogonomyrmex* (*Pogonomyrmex barbatus*; XP_011636912.1), *Papilio* (*Papilio xuthus*; BAM17768.1)

The CDS of *D. gallinae Der g 11* (Accession number LT555400) was 2,622 bp, encoding an 874 amino acid sequence. The sequence with highest homology to Der g 11 was from another member of the order Mesostigmata, *M. occidentalis* with an amino acid identity of 95 %. The predicted protein, Der g 11, also had homologues in the tick species *R. microplus*, *H. longicornis* and *Ixodes scapularis*, with amino acid identities of 84, 84 and 82 %, respectively whereas the amino acid identities of Der g 11 with the orthologous proteins from astigmatid mites were lower at 76 % for *Sarcoptes scabiei* and 75 % for *P. ovis* (Fig. 1).

**Fig. 2:**
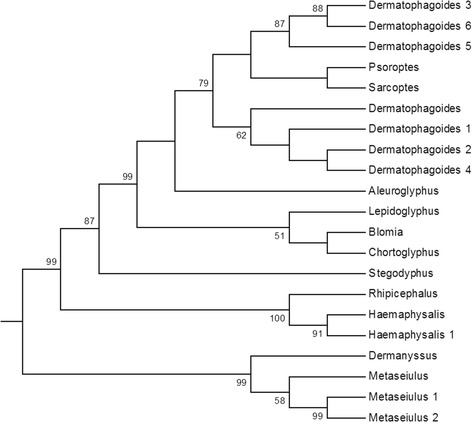
Relationships between selected sequences of Der g 10 from different invertebrate species. The neighbour-joining tree was bootstrapped 1,000 times using Clustal X and the resulting tree viewed with TreeView. Each node is annotated with a figure indicating the degree of bootstrap support for each branch. Abbreviations and accession numbers are as follows: *Dermanyssus* (*Dermanyssus gallinae*; CAJ44440.1), *Metaseiulus* (*M. occidentalis*; XP_003745223.1), *Metaseiulus* 1 (*M. occidentalis*; XP_003745222.1), *Rhipicephalus* (*R. microplus*; O97162.1), *Haemaphysalis* (*H. longicornis*; Q8IT89.1), *Haemaphysalis* 1 (*Haemaphysalis qinghaiensis*; ABQ96858.1), *Blomia* (*B. tropicalis*; ABU97466.1), *Lepidoglyphus* (*Lepidoglyphus destructor* (Q9NFZ4.1)), *Psoroptes* (*P. ovis* (CAJ38272.1)), *Dermatophagoides* (*D. farinae*; BAA04557.1), *Dermatophagoides* 1 (*D. farinae*; Q23939.2), *Dermatophagoides* 2 (*D. farinae*; ABU97468.1), *Dermatophagoides* 3 (*D. pteronyssinus*; ACI32128.1), *Chortoglyphus* (*Chortoglyphus arcuatus*; AEX31649.1), *Sarcoptes* (*S. scabiei*; AFH08744.1), *Stegodyphus* (*S. mimosarum*; KFM56635.1), *Dermatophagoides* 4 (*D. farinae*; AIO08865.1), *Dermatophagoides* 5 (*D. pteronyssinus*; AAB69424.1), *Dermatophagoides* 6 (*D. pteronyssinus*; O18416.1), *Aleuroglyphus* (*Aleuroglyphus ovatus*; AAX37287.1), *Metaseiulus* 2 (*M. occidentalis*; XP_003745225.1)

The top two hits when the nucleotide sequence for Der g 10 was searched against the NCBI nr database using Blastx were two isoforms of tropomyosin from *M. occidentalis* with a 97 and 94 % identity to Der g 10, respectively. The next two species with closest homologues were again the ticks *R. microplus* and *H. longicornis* with 89 and 88 % identity, respectively. As with Der g 11, homologues from the astigmatid mite species had lower amino acid identities to Der g 10 with *P. ovis* and *S. scabiei* tropomyosins being 86 % identical (Fig. 2).

Overall, tropomyosin and paramyosin sequences from *D. gallinae* were both most closely related to orthologues from species belonging to the order Mesostigmata. Phylogenetic analysis placed Der g 10 and 11 into discrete clades with the *M. occidentalis* orthologues (Figs. 1 and 2), with a stronger inferred relationship with the tick species compared to the astigmatid mites, which partitioned separately.

### Anatomical distribution of Der g 11 and Der g 10 in *D. gallinae*

**Fig. 3:**
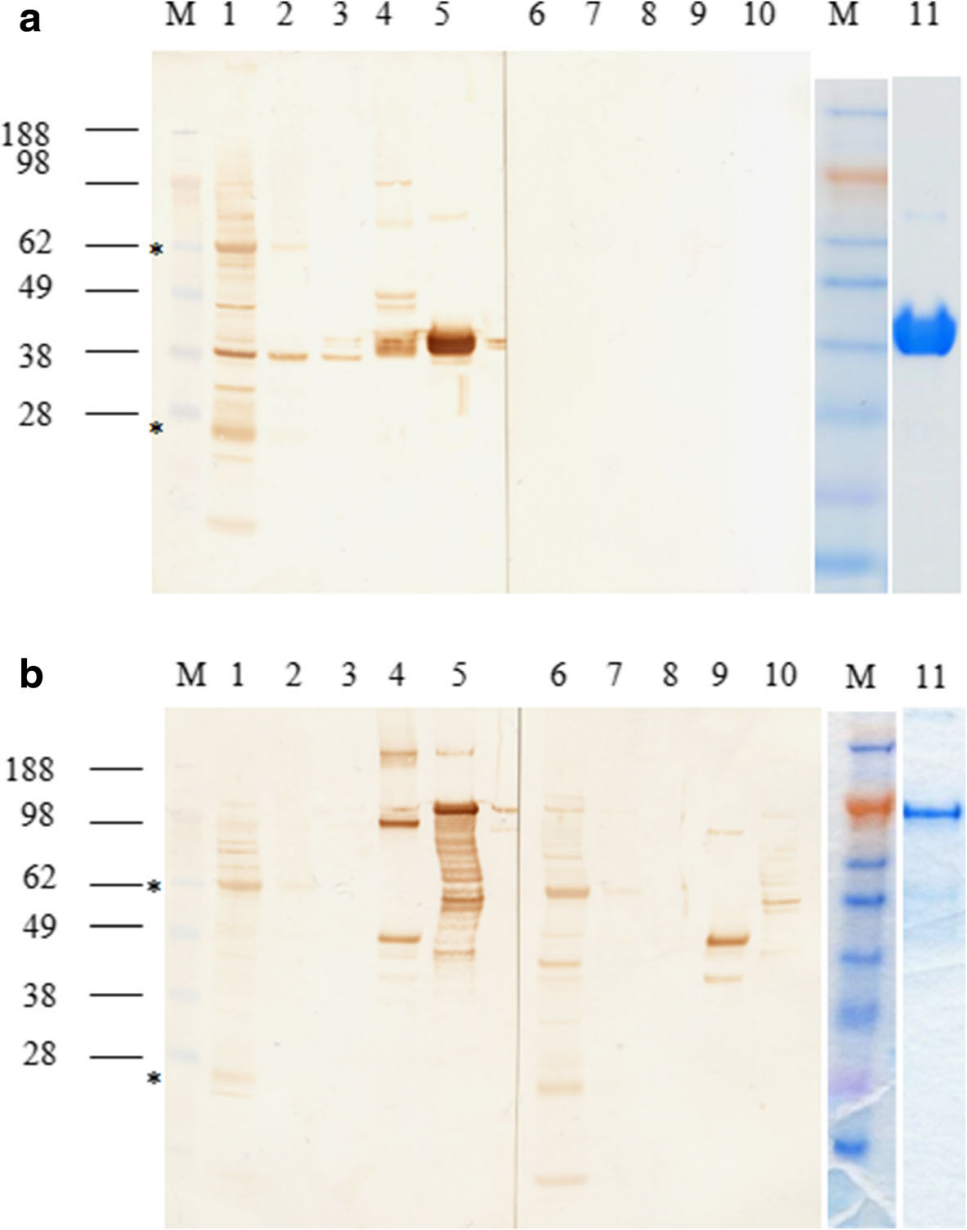
Immunoblots of native and recombinant Der g 10 and Der g 11 from *Dermanyssus gallinae*. **a** Tropomyosin. **b** Paramyosin. For both immunoblots: Lanes 1 and 6: Water soluble proteins; Lanes 2 and 7: Membrane associated proteins; Lanes 3 and 8: Integral membrane proteins; Lanes 4 and 9: Insoluble proteins; Lanes 5 and 10: Recombinant protein; Lane 11: Recombinant protein. Lanes 1–5 were probed with IgY extracted from egg yolk from hens immunised with either recombinant Der g 10 (**a**) or Der g 11 (**b**), and Lanes 6–10 were probed with control sera. Lane 11 was stained with SimplyBlue safe stain (Invitrogen). Lane M: SeeBlue plus 2 molecular mass markers, in kDa (Invitrogen). Stars indicate heavy and light chains of IgY in Lanes 1 and 2 for **a**, and 1, 2, 6 and 7 for **b**

Immunoblots probed with IgY antibodies raised against the recombinant forms of Der g 10 and Der g 11 demonstrated the presence of Der g 10 in PBS-soluble, membrane-associated, integral-membrane and urea-soluble protein preparations from the mite as well as specific recognition of the recombinant bacterially-expressed Der g 10 (Fig. 3a). In contrast, Der g 11 was present only in the urea-soluble fraction of the native proteins (Fig. 3b). The recombinant form of Der g 11 was specifically bound by anti-Der g 11 IgY. The heavy and light chains of IgY were also detected in the PBS-soluble and membrane-associated proteins extracted from mites at a molecular weight of 65 kDa and 25 kDa, respectively, on both immunoblots. Lane 11 in Fig. 3a and b show the two recombinant proteins that were used to immunise the hens.

**Fig. 4:**
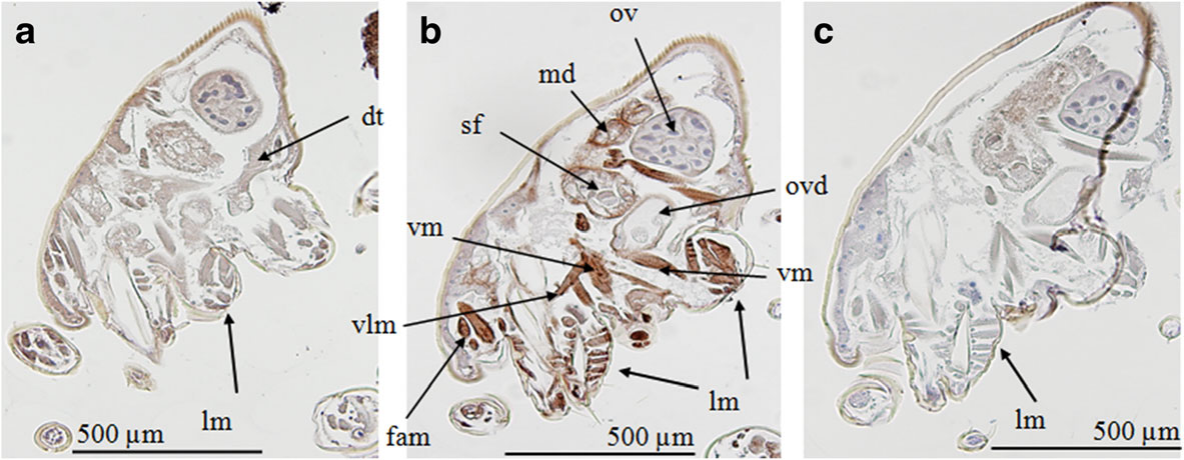
Immunolocalisation of Der g 10 and Der g 11 in sections of *Dermanyssus gallinae*. **a** Der g 11. **b** Der g 10. **c** No primary antibody control slide. *Abbreviations*: dt, digestive tract; fam, feeding associated muscles; lm, locomotory muscle bundles; md, midgut diverticulum; ov, ovaries; ovd, oviduct; sf, *sacculus foemineus*; vlm, ventral longitudinal muscle; vm, ventral muscle. Brown staining represents presence of protein

Immunolocalisation of Der g 10 and Der g 11 demonstrated specific staining of both proteins throughout the sections in multiple mite tissues, reflecting their roles in muscle movement and tissue structural integrity (Fig. 4). For both proteins, there was a high intensity of staining present in the locomotory muscle bundles compared to the staining observed in control sections [40].

### Effects of ingesting IgY specific for Der g 10 and Der g 11 on *D. gallinae* mortality

**Fig. 5:**
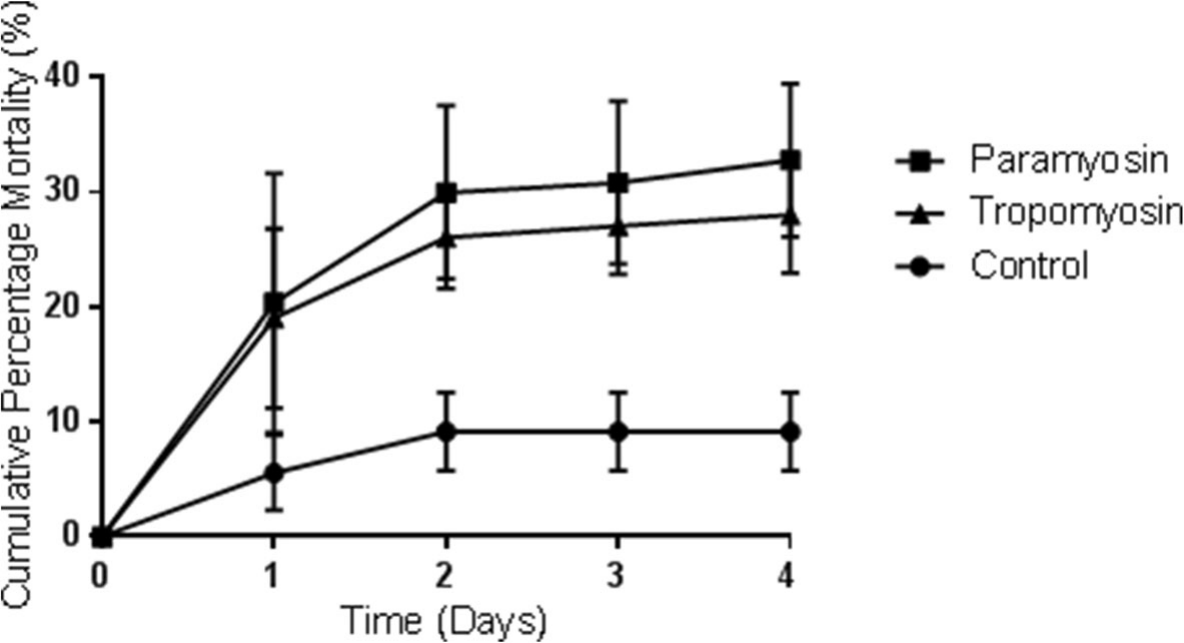
Mortality of *Dermanyssus gallinae*. Fed heparinised chicken blood enriched with antibodies against Der g 10 or Der g 11. Mortality was monitored daily over a 4-day period following feeding. Each point represents cumulative mean percentage mortality and was derived from data recorded in three independent experiments. The error bars represent the standard error of the mean (SEM)

The vaccine potential of recombinant Der g 10 and Der g 11 was investigated by feeding antibodies against the proteins to *D. gallinae* in vitro. Mites were fed a blood meal enriched with 2 mg/ml of IgY generated against Der g 10, Der g 11 or IgY from non-immunised hens to serve as a control. The majority of *D. gallinae* mortality occurred within the first 2 days after feeding (Fig. 5), a mite was considered dead if it had desiccated. The mite mortality at 24 h was 20 % (standard error of the mean (SEM) = 11.3 %) for Der g 11 and 19 % (SEM = 7.8 %) for Der g 10, compared to 6 % (SEM = 3.3 %) mortality observed in the control group. A further increase in mortality of 10 % for Der g 11 and 7 % for Der g 10 was seen from 24 to 48 h, with a reduction in variability between mite groups (SEM reduced to 7.5 and 4.4, respectively). In comparison, the increase in mortality in the control group was lower at 3 % (SEM = 3.4) during the same 24 to 48 h time period. Little mortality occurred after the 48 h time point for any of the groups.

The cumulative mortality of *D. gallinae* at 96 h after feeding on either anti-Der g 10 or anti-Der g 11 antibodies was statistically significantly higher than that of the control groups [*t* = 4.34, *df* = 22, *P* < 0.001 for anti-Der g 10 (19 % mortality); *t* = 2.89, *df* = 22, *P* = 0.009 for anti-Der g 11 (23 % mortality)].

## Discussion

Here we have characterised *D. gallinae* allergens paramyosin (Der g 11), tropomyosin (Der g 10) and evaluated their potential as vaccine candidates. We have shown that recombinant versions of the two muscle-related proteins can generate IgY responses in hens and that when these antibodies were fed to *D. gallinae*, significant mortality ensued. Identifying a reliable, affordable and easy to administer control method for *D. gallinae* would improve the welfare of hens and poultry workers, increase egg production and reduce costs to the egg industry. In addition, a safe control strategy for *D. gallinae* that does result in potentially toxic residues in the food chain is desirable and vaccination fulfils all of those requirements. However, vaccination would not produce sterile immunity in the chickens, vaccination would be used as part of an integrated control strategy that would also include emerging techniques [8].

In recent years, several vaccine candidates have been identified in *D. gallinae* using a pragmatic approach employing successive rounds of native protein fractionation and testing and/or by selecting antigens by their inferred orthology with protective antigens from other parasite species [11, 20, 27, 31, 36, 37, 39, 41–44]. Many of the recombinant *D. gallinae* antigens or native antigen extracts previously tested for vaccine efficacy using the in vitro feeding device [36] have produced a statistically significant increase in *D. gallinae* mortality after one blood meal. Table 1 summarises the net increase in mite mortality 48 h after in vitro feeding on blood enriched with antibodies raised against the listed *D. gallinae* recombinant proteins or native protein extracts that have been tested by our group*.*

**Table 1:** Poultry red mite mortality 48 h after feeding on blood enriched with antibodies. Values shown are for all recombinant proteins and native protein extracts evaluated at Moredun Research Institute thus far. Data were normalised by subtracting the percentage mortality of the mites in the control groups from the mortality of the mites in the test groups

Vaccine antigen	Antigen type	Increase in percentage mortality at 48 h after feeding (%)	Reference
Whole SME	Native	24.0*	[20]
IEX-Group-4	Native	23.5*	[20]
Deg-VIT-1	Recombinant	21.9*	[20]
Der g 11	Recombinant	20.1*	Present study
IEX-Group-1	Native	19.5*	[20]
Deg-HGP-1	Recombinant	18.9*	[20]
Deg-PUF-1	Recombinant	18.4*	[20]
Der g 10	Recombinant	16.5*	Present study
Deg-CPR-1	Recombinant	14.5	[20]
IEX-Group-3	Native	13.0*	[20]
Deg-SRP-1	Recombinant	12.3*	[20]
IEX-Group-5	Native	11.4*	[20]
PBS-soluble	Native	10.1*	[36]
Dg-CatD-1	Recombinant	6.9*	[39]
Deg-ASP-1	Recombinant	5.6	[20]
Dg-HRF	Recombinant	4.1*	[37]
Deg-GPD-1	Recombinant	4.1	[20]
Deg-PUF-3	Recombinant	3.5	[20]
Dg-CatL-1	Recombinant	2.6*	[39]
Membrane associated	Native	2.2	[36]
Deg-PUF-2	Recombinant	0.6	[20]
Urea- soluble	Native	0.2	[36]
Integral membrane	Native	-1.5	[36]
IEX-Group-2	Native	-4.2	[20]
Deg-SRP-2	Recombinant	-8.2	[20]

*Statistically significant difference (*P* < 0.05) between mortalities in the control and antigen test groups in the original study

In comparison with the net mortality induced by other vaccine candidates listed in Table 1, Der g 11 had the fourth highest increase in mortality overall and the second highest mortality attributable to immunisation with a recombinant protein. Der g 10, which had a net increase in mortality of 18.9 %, was the eighth highest overall and the fifth highest recombinant protein. Therefore, Der g 11 is one of the best performing recombinant vaccine candidates tested to date in terms of inducing *D. gallinae* mortality.

When a recombinant form of tropomyosin from the astigmatid mite *S. scabiei* was used to immunise rabbits there was an increase in serum levels of antigen-specific IgG after immunisation. However, there was no apparent effect on mite mortality as the infested areas on the rabbits increased rapidly and mites were detected in skin scrapings from both the control groups and the vaccinated group [45]. Similarly, when a recombinant version of *H. longicornis* tropomyosin was injected into rabbits there was no impact on tick mortality following challenge but immunisation with the recombinant *H. longicornis* tropomyosin did significantly reduce the engorgement weight (150 mg compared to 186 mg in the control; *P* < 0.05), egg laying rate (6 % compared to 56 % in the controls; *P* < 0.05) and egg hatching, which was totally blocked (0 % after vaccination) of ticks feeding on the immunised hosts [27]. These reproduction-associated indicators of vaccine efficacy were not measured in the current study but with further refinement of the in vitro feeding assay they could be incorporated into future vaccine efficacy studies.

In vaccine studies with other parasites, using paramyosin and tropomyosin as antigens, a 34 % reduction in *A. viteae* adult worm burden was recorded when tropomyosin was used to immunise jirds [41] and reductions of *S. japonicum* burden of between 23 and 40 % have been recorded when paramyosin was used to immunise mice [28]. Paramyosin from the liver fluke *Chlonorchis sinensis* was also used in a vaccine study where a recombinant version of the protein was used to immunise rats, which were then challenged with metacercariae. The faecal egg counts (51 % of those measured in controls) and worm burdens (54 % of those measured in the controls) were significantly reduced by immunisation [42]. When a recombinant version of *Dictyocaulus viviparus* paramyosin was used to vaccinate cattle it caused a 52 % average reduction in larval shedding and a 44 % reduction in worm burden over two experiments [43].

These studies all used endo-parasitic organisms that are continually under pressure from the host immune system. The in vitro feeding assay used herein for an ectoparasitic organism only offers *D. gallinae* blood for a single night, which equates to one blood meal. During the course of the *D. gallinae* life-cycle, a mite will feed multiple times [46], which would frequently expose it to the host immune system. The percentage mortality observed could therefore increase as *D. gallinae* feed multiple times, potentially leading to impact on *D. gallinae* populations in a field setting. In addition, as the mortality of *D. gallinae* fed anti-Der g 10 and anti-Der g 11 antibodies individually was relatively low these, or other antigens, may be combined to produce a cocktail vaccine whose components could act additively or synergistically to produce a higher level of mortality (e.g. see [47]).

The mechanisms of action behind the mortality-inducing effects of ingested anti-Der g 10 and anti-Der g 11 are not yet understood but, for both proteins, antibody-binding to mite tissues may inhibit the functions of the proteins, leading to disruption of cytoskeletal function or formation [47]. Evidence has also been found that paramyosin, from *Trichinella spiralis,* can sequester host C9, reducing cell damage to the parasite from the host complement cascade, so a vaccine based on paramyosin may prevent the parasite from subverting the host immune response [48]. Both of these actions would ultimately cause cell death and the breakdown of *D. gallinae* gut. This breakdown of the gut could explain the characteristic “red leg” phenotype that can be seen in some of the dead *D. gallinae* observed [18].

## Conclusions

The experiments described in this paper have shown that tropomyosin and paramyosin are present in *D. gallinae* and, when they were used as immunising antigens in hens an antibody response was generated against them, which had a statistically significantly deleterious effect on the survival of *D. gallinae*. Therefore, these antigens should be considered as potential vaccine candidates against *D. gallinae*, the poultry red mite*.*

## Abbreviation

PRM: Poultry red mite
